# Identification of the Molecular Subgroups in Idiopathic Pulmonary Fibrosis by Gene Expression Profiles

**DOI:** 10.1155/2021/7922594

**Published:** 2021-10-04

**Authors:** Ning Zhang, Yali Guo, Cong Wu, Bohan Jiang, Yuguang Wang

**Affiliations:** Department of Respiratory Medicine, Beijing Hospital of Traditional Chinese Medicine, Affiliated to Capital Medical University, Beijing, China

## Abstract

**Background:**

Idiopathic Pulmonary Fibrosis (IPF) is one of the most common idiopathic interstitial pneumonia, which can occur all over the world. The median survival time of patients is about 3-5 years, and the mortality is relatively high.

**Objective:**

To reveal the potential molecular characteristics of IPF and deepen the understanding of the molecular mechanism of IPF. In order to provide some guidance for the clinical treatment, new drug development, and prognosis judgment of IPF. Although the preliminary conclusion of this study has certain guiding significance for the treatment of IPF and so on, it needs more accurate analytical approaches and large sample clinical trials to verify.

**Methods:**

220 patients with IPF were divided into different subgroups according to the gene expression profiles, which were obtained from the Gene Expression Omnibus (GEO) database. In addition, these subgroups present different expression forms and clinical features. Therefore, weighted gene coexpression analysis (WGCNA) was used to seek the differences between subtypes. And six subgroup-specific WGCNA modules were identified.

**Results:**

Combined with the characteristics of WGCNA and KEGG enrichment modules, the autophagic pathway was only upregulated in subgroup I and enriched significantly. The differentiation pathways of Th1 and Th2 cells were only upregulated and enriched in subgroup II. At the same time, combined with clinical information, IPF patients in subgroup II were older and more serious, which may be closely related to the differentiation of Th1 and Th2 cells. In contrast, the neuroactive ligand-receptor interaction pathway and Ca^+^ signaling pathway were significantly upregulated and enriched in subgroup III. Although there was no significant difference in prognosis between subgroup I and subgroup III, their intrinsic biological characteristics were very different. These results suggest that the subtypes may represent risk factors of age and intrinsic biological characteristics and may also partly reflect the severity of the disease.

**Conclusion:**

In conclusion, current studies have improved our understanding of IPF-related molecular mechanisms. At the same time, because the results show that patients from different subgroups may have their own unique gene expression patterns, it reminds us that patients in each subgroup should receive more personalized treatment.

## 1. Introduction

Idiopathic Pulmonary Fibrosis (IPF) is a chronic, progressive, irreversible, and usually fatal interstitial lung disease with unknown etiology and histopathological manifestations of usual interstitial pneumonia (UIP) [1]. The prevalence and incidence rate of IPF are not yet clear, but the trend is increasing year by year, and the mortality rate is also on the rise [2]. The possible risk factors for IPF include smoking, environmental exposure, microbial factors, genetic factors, and gastrointestinal diseases [3]. The main clinical symptoms of IPF are cough, progressive dyspnea, fatigue, and so on [4, 5]. The clubbing fingers could be found in the clinical physical examination, and crackles could be heard in auscultation.

With the development of high-throughput sequencing technology and microarrays, it provides a good opportunity to further understand IPF. Wang et al. [6] analyzed IPF-related genes based on microarray data through gene set enrichment analysis (GSEA) and differentially expressed genes (DEGs) analysis and integrated 3 public microarray data sets, including 54 IPF samples and 34 normal samples. The results showed that there are 350 genes in DEGs related to IPF. Gene Ontology (GO) and Kyoto Encyclopedia of Genes and Genomes (KEGG) enrichment analyses indicated that inflammatory response, smooth muscle cell proliferation, and chemokine-mediated signaling pathways may be potential targets for IPF therapy. These results may be beneficial to the development of IPF diagnosis and treatment strategies. Fan et al. [7] used microarray data which were downloaded from the GEO database to comprehensively analyze the relationship between bioinformatics and IPF. The results show that there are 67 differentially expressed genes in the three IPF gene expression profile data sets involved and that may participate in the progression of IPF disease by participating in cell adhesion, bioadhesion, extracellular matrix-receptor interaction, and focal adhesion. Wang et al. [8] downloaded GSE49072 gene expression profile from the GEO database and performed a series of bioinformatics analysis (including GO and KEGG enrichment analysis, functional annotation, and protein interaction (PPI) network construction on the String website). The final results showed that 551 DEGs were detected, including 205 downregulated and 346 upregulated. Among the upregulated genes, the expression of secretory phosphoprotein 1 and platelet basic protein is the most significant. At the same time, DEGs in the mitogen-activated protein kinase (MAPK) signaling pathway and chemokine signaling pathway play an important role in the occurrence and development of IPF. Microarray technology is a new high-throughput technology, which is changing the way we study biology. It can be seen that microarray data is the result of high-throughput sequencing. Through the analysis of microarray data, we can screen out the differentially expressed genes of diseases and even obtain the differentially expressed core genes, which is conducive to the diagnosis and treatment of diseases [9, 10]. For example, by analyzing the relevant microarray database, Udhaya Kumar et al. [11] identified seven core genes associated with familial hypercholesterolemia, and these genes may increase the risk of atherosclerosis. Thus, it is beneficial to the development of new drugs and the treatment of diseases. After analyzing the microarray database of lung squamous cell carcinoma, Fu et al. [12] extracted differentially expressed genes related to immunity and constructed the immune signal based on IRG, which has certain guiding significance in judging the progress and prognosis of the disease. Microarray data analysis can screen out the DEGs of diseases and link the differentially expressed genes with specific biological functions, which is conducive to understanding diseases from the molecular level and plays an important role in clinical treatment and new drug development [13].

With the cost reduction of high-throughput sequencing technology and the development of bioinformatics, increasing researchers will use high-throughput technology and bioinformatics to reveal the pathogenesis of IPF. However, most studies only focus on the differences between patients with IPF and normal controls, but little attention is paid to the differences between patients with IPF. In cancer research, to reveal the heterogeneity between tumors, guide treatment, and judge prognosis, tumor samples are usually divided into several subtypes according to gene expression patterns [14]. Peng et al. [15] conducted subcomponent analysis of 352 patients with coronary heart disease according to gene expression spectra, revealed the potential molecular characteristics of different types of coronary artery disease (CAD), enhanced the understanding of CAD molecular mechanisms, and had certain guiding significance for the clinical treatment of CAD. To enhance our understanding of the molecular mechanism of IPF, we also classified the cases of IPF into subgroups according to the relevant gene expression profiles and analyzed them through a series of bioinformatics methods, annotating the corresponding coexpression function modules to reveal the characteristics of each subgroup. Specifically, each subtype showed different expression patterns and disease severity.

## 2. Materials and Methods

### 2.1. Data Collection

#### 2.1.1. Download Data

The Gene Expression Omnibus (GEO) website (https://www.ncbi.nlm.nih.gov/geo/) archives and distributes free microarray, next-generation sequencing (NGS), and other forms of high-throughput functional genomic data [16]. The GEO website was entered, “idiopathic pulmonary fibrosis” was input in the web page, and the GEO data set database was selected. After entering the search results page, the series option was checked, and the expression profiling by array was selected. This study is Homo sapiens. Then, gene chips with more than 40 samples were included in this study, and their platform files and sequence probe matrix files were downloaded, respectively.

#### 2.1.2. Annotation of GEO Data

Programming language Perl (http://www.perl.org/) is known as “Swiss chainsaw of programming language”; it is an excellent choice for developing microarray data processing solutions [17]. In this study, Perl software will be used to extract and sort out the downloaded gene chip-related content, including gene expression matrix, clinical characteristics, and probe set. The platform file downloaded from the GEO website is processed with Perl software to obtain a text with the row name of the gene name and the column name of the sample name. And the new file is the corresponding gene chip name as the data for subsequent research. The information about clinical features in the probe matrix file was extracted into the newly created Excel as the clinical data file for the research.

### 2.2. Elimination of Batch Effect

Firstly, “limma” package and “sva” package of the R/bioconductor package are used to merge the expression data [18]. When the data are combined, the mean value is taken for the data with multiple lines of one gene, and only one line is reserved. For the data with a large value, log2 is taken for conversion. Because the integrated microarray data come from different gene chips, it is necessary to eliminate the “batch effect” to eliminate the cumulative error caused by time, location-related experimental changes, and so on [19]. Based on the systematic review, combat was able to identify more true and false positives. Meanwhile, the combat method could be used to normalize the expression values from different batches or platforms [20]. Therefore, we choose the combat method to eliminate the batch effect between the two platforms. Finally, the R/ggplot2 package was used to analyze the main component to evaluate whether the batch effect was removed [21].

### 2.3. Consensus Clustering

The “limma” package and “consensus cluster plus” package of the R/bioconductor package were used for consensus clustering, which classified IPF cases into different subgroups [22]. The *K*-means algorithm with Spearman distance was used for clustering [23]. The maximum cluster number was set to 10, and the final cluster number was determined by the consistency matrix and cluster consistency score (>0.7).

### 2.4. Comparing the Clinical Characteristics of the Three Subgroups

To obtain the clinical characteristics among the three subgroups, guide the clinical treatment, and judge the prognosis, the clinical characteristics of the three subgroups were compared. Pairwise proportion tests were used to compare the proportion of men in the three subgroups. In addition, pairwise Wilcoxonʼs rank-sum test was used to test whether there were differences in age and GAP models between subgroups. GAP models included gender (G), age (A), and two pulmonary physiological variables (P) (FVC and DLco) [24].

### 2.5. Extraction of Specific Upregulated Genes in Subtypes

To better understand the disease from the aspects of molecular mechanism, screen specific expression genes for follow-up basic research, and guide the clinical treatment and the development of new drugs, the specific upregulated genes of each subgroup were extracted. By comparing the specific subgroup with other subgroups, the specific upregulated genes were identified. It should be noted that Wilcoxonʼs rank-sum test was used to test the differential expression, the corrected threshold was *p* < 0.05, and the absolute difference of means > 0.2. For a given gene, the difference in the mean is calculated by subtracting the average expression of the normal control group from the case of a particular subgroup.

### 2.6. Gene Set Enrichment Analysis

Gene set enrichment analysis (GSEA) was used to observe whether the specific differential genes in each subgroup were also different from normal samples [25]. To better understand the disease from the molecular level, GSEA was implemented in GSEA desktop version 4.1.0 in the GSEA prerank mode. The genome database consisted of subgroup-specific genes. And the gene list of each subgroup was ranked by *p* values using paired Student's *t*-test, which was calculated by comparing the IPF cases of each subgroup with the normal control group.

### 2.7. Weighted Gene Coexpression Network Analysis

Weighted gene coexpression analysis (WGCNA) was used to analyze the specific genes in each subgroup to determine the modules that can represent the biological functions of each subgroup and which could be used to identify candidate biomarkers or therapeutic targets [26]. WGCNA has been proved to be an effective method to detect multiple coexpression modules, which can be used to find clustering (modules) of highly related genes [27]. The optimal power value was found through the power value scatter plot, and the distance between genes was calculated. In addition, the average method and the dynamic method were used for hierarchical clustering analysis; the clustering diagram and the module classification of genes are, respectively, established; and similar modules are merged. We finally determined 6 functional modules. Spearmanʼs correlation coefficients and the corresponding *p* values between clinical features and functional modules were calculated by using the *cor* function of Spearmanʼs method in the WGCNA package. At the same time, the function option of the labeled head map in “limma” and “pheatmap” package was applied to draw the heat map.

### 2.8. KEGG Enrichment Analysis

The upregulated genes in each subgroup of WGCNA were analyzed by KEGG enrichment analysis, to understand the characteristics of each subgroup on a deeper level from the molecular mechanism level, and provide certain guiding significance for clinical treatment and prognosis judgment. The gene group of the KEGG pathway was downloaded from MSigDB, and the gene species was human [28]. In KEGG enrichment analysis, the *p* value filter condition was set to <0.05, and the corrected *p* value filter condition was 1.

## 3. Results

### 3.1. Characteristics of IPF Subjects

Five independent microarray information were included in this study, involving four independent clinical trials. The gene expression data were fetched from the GEO database with accession GSE33566 (David Schwartz et al., 2012; *n* = 123), GSE49072 (Eric Billings et al., 2014; *n* = 84), GSE53845 (Alex Abbas et al., 2014; *n* = 48), GSE70866 (Antje Prasse et al., 2018; *n* = 196), and GSE70867 (Antje Prasse et al., 2018; *n* = 321). GSE33566 and GSE70866 provide clinical information of age. GSE33566, GSE53845, and GSE70866 provide gender clinical information. In addition, GSE33566 and GSE70866 also provide clinical information about DLco and GAP models, respectively, reflecting the severity of the disease to a certain extent.

### 3.2. Removal of Batch Effect by Cross-Platform Normalization

**Figure 1:**
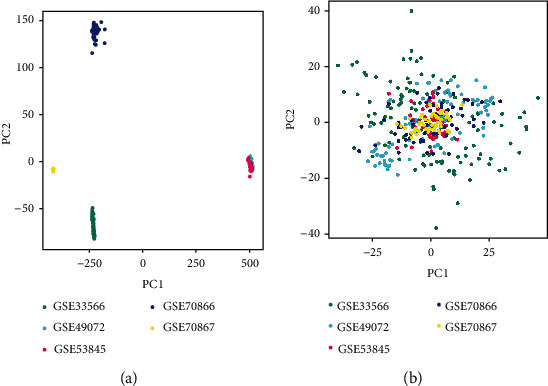
Principal component analysis of gene expression data set. The dots in the scatter plot are based on the first two main components of the gene expression profile (PC1 and PC2) visualization samples: (a) no elimination of batch effect; (b) elimination of batch effect. The colors represent samples from five different data sets.

To remove the batch effect from different platforms and batches, we used the combat method to eliminate the batch effect between data sets. A total of 7959 genes were detected by the two microarray platforms. Before eliminating the batch effect, samples were clustered in batch according to the top two principal components (PCs) of the unnormalized expression values (Figure 1(a)). In contrast, the scatter plot was standardized based on principal component analysis, and the results showed that the batch effect caused by different platforms was clearly removed (Figure 1(b)). The results showed that the batch effect was successfully eliminated by cross-platform normalization.

### 3.3. Consensus Clustering of IPF Cases

**Figure 2:**
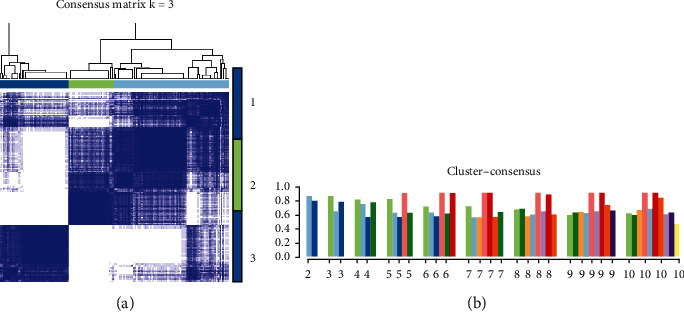
Consensus clustering analysis of gene expression profiles for Idiopathic Pulmonary Fibrosis (IPF) cases. (a) The heat map represents the consensus matrix with a cluster count of 3, which was determined by the minimal consensus scores for subgroups (>0.7). (b) The bar charts represent the consistent score of subgroups with cluster numbers between 2 and 10.

Cluster analysis (an unsupervised clustering method) was carried out by using the batch effect corrected expression file and the sample information of the disease (diagnosed as IPF) group. 220 patients with IPF were divided into subgroups (see Section 2.3). According to the consistency score of data statistics, the gene expression profile was divided into three subgroups by cluster analysis. The number of cases in subgroups I, II, and III was 43, 111, and 66, respectively, which had significantly different expression patterns. On the contrary, based on the consistency matrix, a high degree of similarity in gene expression patterns was observed in each subgroup (Figure 2(a)).

Generally speaking, the higher the consistency score, and the more the group classification, the more robust the subtype. In the results of this study, although the consistency score of the 2 groups was the highest, there were fewer groups. And whether divided into 2 or 3 groups, the consistency score between groups was greater than 0.7. According to the above results, 220 patients with IPF were divided into 3 subgroups (Figure 2(b)).

To describe the clinical features of the three subgroups, the age of GSE33566 and GSE70866 data sets was analyzed; the gender of GSE33566, GSE53845, and GSE70866 data sets was statistically analyzed; and the GAP models in the GSE70866 data set were also statistically analyzed. Due to the lack of DLco data in the GSE33566 data set, no original data was found, so no statistical analysis was made.

**Figure 3:**
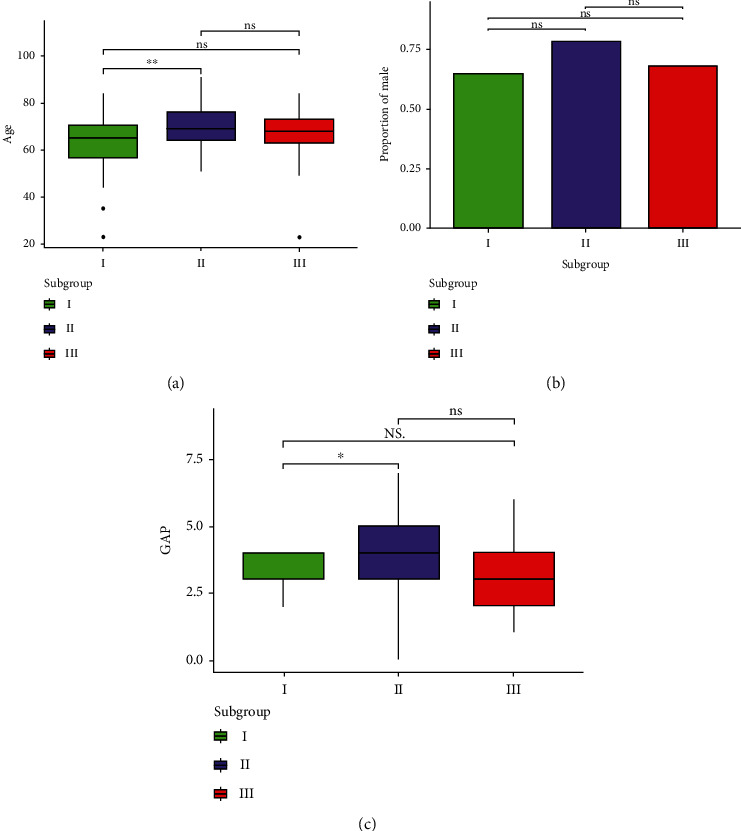
The pairwise comparison of clinical characteristics between the subgroups. Box-chart (a, c) showed the age and GAP models of each subgroup, respectively. (b) The proportion of males in each subgroup is represented by the bar-plot.

The results of age statistics showed that patients in subgroup II were older than those in other subgroups, and there was a significant difference between subgroup I and subgroup II (*p* < 0.001), but there was no significant difference between subgroup II and subgroup III or between subgroup I and subgroup III (*p* > 0.05) (Figure 3(a)).

The results of gender statistics showed that although the proportion of males in subgroup II was higher than that in the other two groups, there was no significant difference in the proportion of males among the three subgroups (*p* > 0.5) (Figure 3(b)).

The GAP model statistical results showed that overall, the gap score of subgroup II was higher than that of the other two groups. In addition, subgroup II was significantly higher than subgroup I (*p* < 0.05) (Figure 3(c)).

In addition, we also analyzed the variance between age and subgroup and found that the subgroup was an independent IPF-related index, which could predict the severity of the disease to a certain extent (Table 1, *p* < 0.05); at the same time, the age of the patient could also predict the severity of the disease to a certain extent, which is consistent with the previous research results (Table 1).

### 3.4. Identification of Gene Coexpression Modules for Each Subgroup

**Figure 4:**
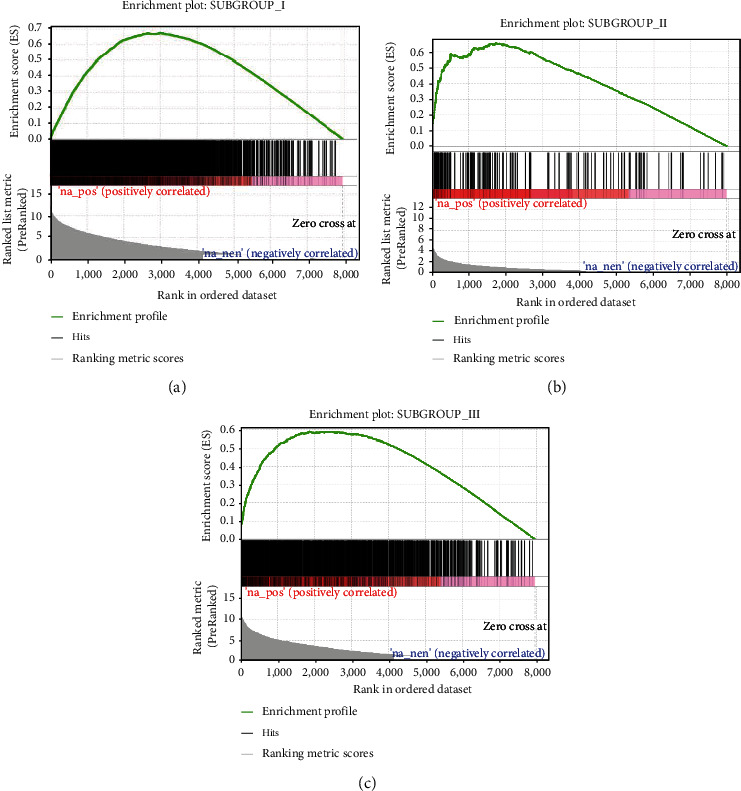
The expression patterns of subgroup-specific upregulated genes. The enrichment plots of (a–c) illustrate that the subgroup-specific upregulated genes are also expressed higher in the corresponding subgroup than the normal controls.

To reveal the gene differences among IPF subgroups, WGCNA was performed at the expression level of specific upregulated genes in each subgroup (see Section 2.7). Pairwise differential expression analysis between every two subgroups identified 2434, 141, and 1329 genes specifically upregulated in subgroups I, II, and III (Benjamin-Hochberg adjusted *p* < 0.05, absolute difference of mean > 0.2). In addition, we compared the gene expression profile of each subgroup with that of the normal control group to analyze the differential expression. GSEA revealed that subgroup-specific upregulated genes were also significantly upregulated in case-control comparison (Figures 4(a)–4(c), FDR < 0.05).

It is worth noting that compared with other subgroups, although subgroup II has the least number of subgroup-specific upregulated genes, its GAP models and age were higher than those of the other two groups, indicating that this group of patients may be more serious. Compared with subgroup III, subgroup I had more upregulated genes, but the GAP models and age were lower. These results suggest that subgroup I may be relatively mild.

**Figure 5:**
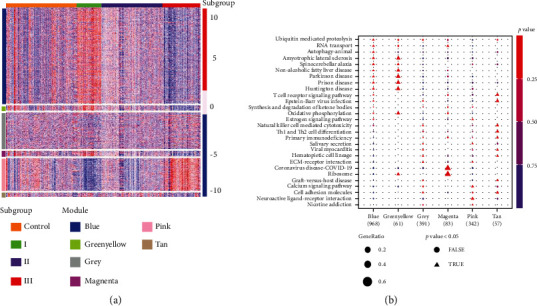
(a) The scaled expression values of genes that comprise each of the six weighted gene coexpression network analysis modules are displayed in the heat map. (b) The gene enrichment analysis of each WCGNA module by the KEGG pathway.

Based on the expression levels of 3906 upregulated genes in the subgroup, a gene expression network was constructed, and six WGCNA modules were identified. The relationship between WGCNA modules and corresponding subgroups is shown in Table 2. The gene enrichment analysis of each WGCNA module by the KEGG pathway showed that the autophagy pathway was only significantly enriched in the blue module, and the oxidative phosphorylation pathway was only significantly enriched in the green-yellow module. The grey module was enriched in the ECM-receptor interaction pathway. The magenta module was enriched in the ribosomal pathway. The pink module was significantly enriched in the neuroactive ligand-receptor (NLR) interaction pathway, and this pathway was only significantly enriched in the pink module. The differentiation pathways of Th1 and Th2 cells were significantly enriched in the tan module. In subgroup I, genes were significantly upregulated in the blue, green-yellow, and magenta modules, and the most upregulated genes were in the blue module. The autophagy pathway was only significantly enriched in the blue modules, including Akt3, PIK3CA, and PIK3R1. In subgroup II, genes were upregulated in the tan module, while Th1 and Th2 cell differentiation pathways were only enriched in this module, including CD247, JAK3, and STAT4. In addition, the genes in subgroup III were significantly upregulated in the pink module, in which the NLR pathway was enriched most significantly, and the NLR pathway was only enriched in the pink module. Combined with the results of Section 3.3, it showed that the subgroup-specific genes could serve as biomarkers independent of these confounding factors and were related to IPF (Table 2, Figures 5(a) and 5(b), and Supplementary Tables 1–3).

### 3.5. Association of Clinical Characteristics and WGCNA Modules

**Figure 6:**
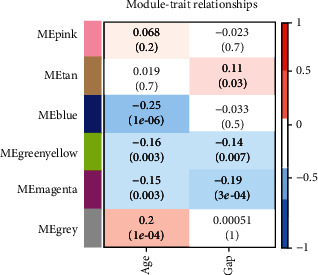
The positive and negative correlation coefficients between WGCNA module and clinical features, GAP models, and age were shown in red and blue, respectively.

To study the relationship between clinical features and WGCNA modules, the correlation coefficients and corresponding *p* values between GAP models or age and eigengenes of each module were calculated (see Section 2). It should be noted that the characteristic genes are represented by the eigenvectors of the gene expression matrix of each module. The results showed that the grey, blue, and pink modules had nothing to do with the GAP models. In contrast, the purplish-red module and green-yellow module were negatively correlated with age and GAP models, and these two modules were significantly enriched in the ribosomal pathway. The tan module was positively correlated with GAP models, while Th1 and Th2 cell differentiation pathways were significantly enriched in the tan module, indicating that immune dysfunction was correlated with GAP models. The grey module was positively correlated with age. The results further showed that the WGCNA module was associated with some clinical features, such as GAP models and age (see Figure 6).

## 4. Discussion

In this study, we analyzed gene expression profiles of IPF cases and normal controls from five independent GEO data sets. The batch effect of different platforms or batches is eliminated. In addition, we successfully divided 220 patients with IPF into three subgroups according to the gene expression profile for the first time. In further analysis, subgroup-specific functional modules or pathways were revealed. Significant associations were observed between clinical features and subtypes. Compared with the other two subgroups, the gap score of subgroup II was higher and the age was older, which indicated that the IPF patients in subgroup II might be the most serious. The consistency clustering based on large sample size and high cluster consistency score (>0.7) showed that our subtype was robust. In summary, the subtypes of IPF are closely related to clinical features and specific functional modules or pathways.

The motivation of this study is the subtype of cancer, which can be identified by gene expression profile or other omics data. In addition, the relationship between subgroup differences and internal or external factors has been widely studied. For example, Kim et al. [29] conducted a subgroup analysis on different types of cancer patients, and the results showed that the low pain/high fatigue subgroup only appeared in the first chemotherapy cycle, and there were significant subgroup differences in pain and fatigue levels at each time point (*p* < 0.05). Seiler et al. [30] explored the ability of molecular subtypes to predict the pathological stage and survival after neoadjuvant chemotherapy (NAC). The results showed that molecular subtypes may affect the benefit of NAC to patients, especially in patients with basal tumors. Jang et al. [31] reported that the molecular stratification of NSCLC transcriptome sequencing data identified different immune molecular subtypes, which predicted the response to programmed cell death 1 blockade. In addition to cancer research, noncancer diseases include Alzheimer's disease, myelodysplastic syndrome, and chronic obstructive pulmonary disease [32–34]. Although these studies have some limitations and confounding factors, they do improve our understanding of the relationship between molecular mechanisms and disease development.

Similar to cancer, rare and complex diseases such as IPF show clinical heterogeneity. Unlike previous studies [7, 35], which only studied the gene expression profiles of patients with IPF or compared with the gene expression profiles of normal controls, we further divided IPF cases into subgroups and revealed that the subjects in different subgroups showed different clinical characteristics. For example, subjects in subgroup II tend to be older and more severe. Although subgroups I and III showed younger age and lighter IPF, subgroup I was probably the least severe. In addition, the proportion of males in subgroup II was significantly higher (about 75%) than that in the previous male IPF epidemiology. Therefore, IPF cases with different clinical features can be clearly distinguished by subtypes. According to the molecular mechanism of action of different subgroups, it has a certain guiding significance for the development of new drugs and treatment of IPF.

Compared with previous studies [36, 37], subgroup-specific functional modules not only confirmed IPF-related regulatory pathways but also linked specific pathways to IPF subjects in specific subgroups or clinical features. For example, it is well known that Th1 and Th2 cell differentiation plays an important role in IPF. The inflammatory response of IPF is considered very similar to the Th2 immune response in T-helper cells, and the polarized T-cell response is thought to play an important role in the development of tissue fibrosis. Th1 cells are involved in phagocyte-dependent inflammation and cell-mediated immunity [38]. And Th1 cells producing interferon and interleukin-12 have been shown to limit the development of tissue fibrosis, while Th2 cells producing interleukin-4 and interleukin-13 have been shown to promote the development of tissue fibrosis [39–41]. The evaluation of lung tissue in IPF patients showed that the expression of Th2 cytokines was higher than that of Th1 cytokines [42]. In our study, in subgroup II, we found that the differentiation pathways of Th1 and Th2 cells were most significantly enriched in this subgroup, indicating that the differentiation of Th1 and Th2 cells is closely related to the age and severity of IPF. The balance of Th1 and Th2 cells plays an important role in autoimmune diseases. It can be speculated that the pathogenesis of subgroup II may be related to autoimmune dysfunction.

However, according to the enrichment results of the KEGG pathway, the main targets involved in Th1 and Th2 cell differentiation include CD247, CD3D, IL2RB, JAK3, and STAT4. CD247 (also known as CD3 chain) is involved in the activation and function of T cells and is one of the susceptibility genes of systemic sclerosis with pulmonary fibrosis [43]. However, whether CD247 is involved in the pathogenesis of IPF and becomes a potential therapeutic target of IPF, we need to carry out relevant basic experiments and prospective clinical trials to verify. JAK and STAT play an important role in IPF. Shi et al. [44] showed that TGF is involved in the pathogenesis of BLM-induced mouse JAKs/STATs pathway. A novel pyrimidine multitarget protein tyrosine kinase inhibitor may be a promising drug for IPF, and one of its mechanisms of action is to inhibit JAK3 kinase [45]. Therefore, JAK-related inhibitors may be one of the main effective drugs for IPF in the future. Because the age and gap score of the patients in subgroup II are higher, and their condition is more serious, we can consider using JAK-related inhibitors and immunomodulators to regulate Th1 and Th2 cells, to improve the patient's condition and improve the quality of life. However, these are preliminary conclusions based on previous research results, which need to be verified by relevant basic experiments and large sample clinical trials.

In contrast, although there were no significant differences in age and GAP models between subgroup I and subgroup III, their intrinsic biological characteristics showed significant differences. In subgroup I, most genes were upregulated in the blue module, while the autophagy pathway was only enriched in this module. Relevant studies have shown that autophagy reduces the pathological process of IPF by regulating fibroblast apoptosis and alveolar epithelial cell aging, and its defects may be involved in the pathogenesis of IPF [46, 47]. According to the enrichment results of the KEGG pathway, autophagy-related pathway targets mainly include Akt3, PIK3CA, and PIK3R1. Recent studies have shown that the PI3K/Akt signaling pathway can regulate mTOR, a target of autophagy [48–51]. Therefore, PI3K-related inhibitors may be more effective in subgroup I patients. However, a large sample of clinical data is needed to verify.

In subgroup III, significant enrichment in neuroactive ligand-receptor interaction and Ca+ signaling pathway was observed. Relevant studies have shown that Ca+ signal transduction plays an important role in promoting the proliferation, transformation, and collagen synthesis and inhibiting the apoptosis of lung fibroblasts [52]. Activation of the Ca+ signaling pathway can increase the sensitivity of cough, so it can be speculated that cough symptoms may be more prominent in subgroup III. According to KEGG pathway enrichment results, calcium signaling pathway-related pathway targets mainly include NTRK2 and P2RX3. In recent years, there is evidence that NTRK2 (also known as TrkB) plays an important role in the neurotrophin receptor tyrosine kinase family [53]. At the same time, TrkB is closely related to the pathogenesis of neurogenic cough [54]. Relevant studies also show that the BDNF/TrkB axis plays a role in EMT promoting the acquisition of IPF (myo) fibroblast phenotype. Targeting BDNF/TrkB is a feasible method to prevent EMT-dependent pulmonary fibrosis [55]. Therefore, for subgroup III patients, the application of TrkB targeted drugs might have better effect. However, it also needs a large number of basic experiments and prospective clinical trials to verify.

In summary, these results further prove that the subtypes represent the development stage and intrinsic biological characteristics of IPF. Similar to the subtypes in cancer, future IPF research should also introduce multiomics data to reveal more accurate molecular subgroups of IPF. However, the analysis of IPF in omics is relatively less. Inspired by the study of cancer subtypes and the subgroup analysis of CAD by Peng et al. [15], we applied a similar strategy to reveal the molecular subgroup of IPF. Current studies have improved our understanding of IPF-related molecular mechanisms. At the same time, because the results show that patients from different subgroups may have their own unique gene expression patterns, it reminds us that patients in each subgroup should receive more personalized treatment. There are limitations to this study. Firstly, although our findings suggest that IPF cases from different subgroups may have different expression patterns, they are based on previous studies. Secondly, although the molecular subtypes of IPF obtained in the preliminary screening of this study have certain guiding significance for development of new drugs, clinical treatment, and prognosis judgment, more rigorous analysis methods and a larger population are needed for prospective verification.

## Figures and Tables

**Table 1:** Analysis of variance for classification of subgroups, age, and their interactions.

	Df	Sum square	Mean square	F value	Pr (>F)
Subgroup	2	10.42	5.212	3.636	0.032^∗^
Age	1	28.30	28.927	19.851	3.88e-05^∗∗∗^
Subgroup : age	2	6.35	3.176	2.228	0.117
Residuals	58	82.68	1.425		

Note. Df: degree of freedom. Significant codes: “∗∗∗” 0.001, “∗∗” 0.01, “∗” 0.05, “.” 0.1, “ ” 1.

**Table 2:** The number of differentially expressed genes by case-control and case-case comparisons and weighted gene coexpression analysis modules in each subgroup.

Subtypes	The specific genes were compared with the normal group	The specific genes were compared with each subgroup	Specific upregulated genes in subgroup	Modular
I	3549	4142	2434	Blue, green-yellow, magenta
II	178	311	141	Tan
III	2567	3786	1329	Pink

## Data Availability

The data used to support the findings of this study are included within the article.

## Supplementary Materials

Supplementary Materials There are three supplementary materials in this article.
