# Supplementary material for: Identification of the Molecular Subgroups in Idiopathic Pulmonary Fibrosis by Gene Expression Profiles
Source: Comput Math Methods Med. 2021 Oct 4;2021:7922594. doi: 10.1155/2021/7922594 (PMC8505108; doi:10.1155/2021/7922594)
Supplement: Supplementary Materials — There are three supplementary materials in this article. [file 7922594.f1.zip › supplementary table 1.pdf]

supplementary table 1

common DEGs between datasets

ID	logFC	P.Value	adj.P.Value
PLA2G7	-1.315573148	3.67E-28	4.86E-25
VCAN	-1.029350431	3.90E-26	4.43E-23
LRRN3	1.329869933	1.25E-24	1.02E-21
SORL1	-1.291277197	1.06E-22	4.67E-20
GSN	-1.41641199	1.38E-22	5.76E-20
LHFPL2	-1.077127017	1.51E-22	5.99E-20
SPP1	-1.537285066	7.53E-22	2.49E-19
EMG1	1.127538641	2.37E-21	7.24E-19
CTSK	-1.218441005	3.11E-21	8.82E-19
TMEM204	1.32962955	2.96E-20	4.50E-18
TGFBI	-1.007035175	3.07E-20	4.50E-18
AQP9	-1.086886818	3.12E-20	4.50E-18
LITAF	-1.064610128	5.82E-20	7.71E-18
EMP1	-1.028878517	6.44E-20	8.40E-18
LYN	-1.172407112	7.26E-20	9.16E-18
ICAM3	-1.140785803	1.57E-19	1.71E-17
S100P	-1.137900626	1.59E-19	1.71E-17
ITGAX	-1.125578779	2.91E-19	2.83E-17
RPL23AP32	1.744771717	3.18E-19	3.01E-17
CD97	-1.098003672	3.38E-19	3.12E-17
CXCR4	-1.289602424	3.89E-19	3.47E-17
PSAP	-1.349222346	4.45E-19	3.93E-17
FAM153A	1.419611385	6.39E-19	5.30E-17
VASP	-1.017590836	6.66E-19	5.36E-17
VIM	-1.447655894	9.69E-19	7.41E-17
LIPA	-1.105319304	1.10E-18	8.28E-17
ARPC5	-1.089586628	1.50E-18	1.06E-16
SPRR1A	-1.047003542	1.64E-18	1.14E-16
TNFAIP2	-1.470952433	2.47E-18	1.55E-16
GCA	-1.014023154	4.81E-18	2.86E-16
SERPINA1	-1.028395308	4.89E-18	2.88E-16
ANKRD2	1.012826369	6.13E-18	3.51E-16
KRTAP5-8	1.752801552	6.41E-18	3.61E-16
SRGN	-1.290442853	7.35E-18	4.00E-16
POU3F3	1.217236126	7.39E-18	4.00E-16
LY96	-1.168648492	9.07E-18	4.81E-16
GFAP	1.992893187	9.66E-18	5.09E-16
IL1RN	-1.329319776	1.09E-17	5.69E-16
APOL3	1.090863446	1.26E-17	6.37E-16

GRN	-1.626795095	1.36E-17	6.78E-16
BDH2	1.017558497	2.19E-17	1.01E-15
BAG3	1.04091118	2.65E-17	1.18E-15
TCIRG1	-1.291190335	2.87E-17	1.27E-15
PRCP	-1.227530174	2.98E-17	1.31E-15
C1orf54	-1.034179185	3.27E-17	1.41E-15
BACH2	1.027744815	3.76E-17	1.59E-15
MSX1	1.807385032	4.38E-17	1.82E-15
LGALS1	-1.087369629	4.51E-17	1.87E-15
LGALS3	-1.159101789	5.74E-17	2.27E-15
MS4A1	1.682978358	5.91E-17	2.32E-15
ATP6AP2	-1.012028862	6.97E-17	2.65E-15
PLBD1	-1.472021814	7.25E-17	2.73E-15
ZNF331	1.046786	7.42E-17	2.78E-15
GDNF	1.522478254	1.35E-16	4.71E-15
HEBP2	-1.019647873	1.57E-16	5.33E-15
KLRF1	1.416666294	2.00E-16	6.45E-15
FBP1	-1.46595606	2.06E-16	6.57E-15
CTSA	-1.228263432	2.56E-16	7.94E-15
CD163	-1.424969543	2.90E-16	8.95E-15
PRX	2.062765089	4.48E-16	1.29E-14
RAC2	-1.28376923	4.67E-16	1.34E-14
S100A11	-1.120322877	4.69E-16	1.34E-14
QSOX1	-1.14244305	4.71E-16	1.34E-14
MSN	-1.163281527	6.45E-16	1.75E-14
CAPG	-1.489456669	6.89E-16	1.85E-14
CD79B	1.238861922	7.49E-16	1.97E-14
ABR	-1.163276374	7.84E-16	2.06E-14
PGD	-1.130861315	8.13E-16	2.11E-14
ACP5	-1.015655048	8.77E-16	2.25E-14
SOX17	1.182488862	9.46E-16	2.40E-14
GLA	-1.106197378	1.09E-15	2.68E-14
TCF7	1.122005711	1.35E-15	3.20E-14
CTSZ	-1.042355244	1.71E-15	3.88E-14
CTSW	1.198695545	1.91E-15	4.27E-14
EMILIN2	-1.304554674	2.28E-15	5.01E-14
CTSD	-1.204736737	2.37E-15	5.13E-14
C19orf53	1.042972848	2.47E-15	5.30E-14
MST1	1.516978107	2.50E-15	5.36E-14
GPT	1.283433865	2.58E-15	5.51E-14
HIST1H3C	-1.042022874	2.69E-15	5.71E-14
ARHGDIB	-1.079445365	2.69E-15	5.71E-14
MRPL40	1.051268902	2.91E-15	6.11E-14
H3F3A	-1.331207858	3.21E-15	6.68E-14

CLDN5	1.212369623	3.25E-15	6.74E-14
ZFP36	-1.101592639	3.25E-15	6.74E-14
MS4A4A	-1.127037041	3.26E-15	6.74E-14
HPGD	1.02982117	3.35E-15	6.89E-14
DAZAP2	-1.087889182	3.63E-15	7.40E-14
ALOX5	-1.138123168	3.66E-15	7.43E-14
MTG1	1.02657113	3.94E-15	7.88E-14
KLHDC2	1.202425055	4.24E-15	8.35E-14
CLIC3	1.050489227	4.28E-15	8.39E-14
ATP6V0C	-1.121850526	4.96E-15	9.58E-14
S1PR1	1.020339134	5.17E-15	9.91E-14
ZNF862	1.029632507	5.25E-15	1.00E-13
LCP1	-1.109184541	5.40E-15	1.02E-13
STAT6	-1.099828989	5.69E-15	1.07E-13
COTL1	-1.131504642	6.49E-15	1.20E-13
KRT81	-1.324459254	7.40E-15	1.35E-13
ATP5S	1.050095248	7.56E-15	1.36E-13
HPCA	1.500170153	8.01E-15	1.42E-13
MARCO	-1.823469581	8.55E-15	1.50E-13
FCGR1B	-1.20788177	8.74E-15	1.53E-13
CXCR5	1.42299781	9.15E-15	1.59E-13
VASH1	-1.070840096	9.53E-15	1.64E-13
NPC2	-1.397217546	9.66E-15	1.66E-13
TYMP	-1.323082543	1.04E-14	1.77E-13
SNX2	-1.005781549	1.16E-14	1.93E-13
ALG13	1.036582009	1.20E-14	1.99E-13
APOE	-1.099850089	1.24E-14	2.04E-13
PTCD2	1.208604816	1.25E-14	2.05E-13
RIC3	1.635624724	1.28E-14	2.09E-13
ZNF492	1.304962827	1.33E-14	2.17E-13
SGK1	-1.036935653	1.44E-14	2.31E-13
ACTB	-1.529716317	1.59E-14	2.48E-13
HSP90B1	-1.266439161	1.59E-14	2.48E-13
USP53	1.27029594	1.69E-14	2.62E-13
CSF1R	-1.428627746	1.73E-14	2.67E-13
S100A8	-1.2802341	1.76E-14	2.70E-13
CEBPB	-1.0838711	2.05E-14	3.06E-13
TUBB6	-1.032305977	2.17E-14	3.22E-13
CHRNB2	1.538903834	2.31E-14	3.39E-13
FCER1G	-1.113945273	2.44E-14	3.53E-13
NDUFAF4	1.117677228	2.66E-14	3.82E-13
CNR1	1.127023883	2.87E-14	4.03E-13
PHACTR4	1.27805266	2.89E-14	4.05E-13
CACNB1	1.222657184	2.90E-14	4.05E-13

SH3BGRL3	-1.405086584	3.06E-14	4.26E-13
NKX3-1	1.247353443	3.15E-14	4.37E-13
PCDH9	1.095819972	3.16E-14	4.37E-13
LMNA	-1.205867342	3.32E-14	4.57E-13
IRX5	1.760770537	3.37E-14	4.62E-13
SMA4	1.288749148	3.57E-14	4.86E-13
ABHD10	1.541070289	4.04E-14	5.35E-13
CNTNAP2	2.182837233	4.17E-14	5.48E-13
HLA-B	-1.116862312	4.28E-14	5.61E-13
S100A10	-1.30061098	4.47E-14	5.81E-13
TSPYL2	1.037695804	4.52E-14	5.85E-13
CEBPD	-1.056183437	4.56E-14	5.89E-13
FRMD4A	1.171014196	4.66E-14	6.02E-13
S100A4	-1.090224779	5.08E-14	6.49E-13
CYP2W1	-1.157420578	5.24E-14	6.67E-13
AIF1	-1.480308092	5.79E-14	7.25E-13
CDC42	-1.16057164	6.40E-14	7.91E-13
SNX3	-1.10584827	6.42E-14	7.92E-13
ATP6AP1	-1.152938163	6.62E-14	8.13E-13
GMFG	-1.205760432	6.83E-14	8.36E-13
RNF144A	1.354243491	7.20E-14	8.74E-13
HCLS1	-1.374381577	7.32E-14	8.86E-13
SPTBN1	1.200214759	7.36E-14	8.90E-13
ACADVL	-1.103919457	8.09E-14	9.66E-13
CHMP2A	-1.120500958	8.16E-14	9.71E-13
HMOX1	-1.015942027	8.61E-14	1.02E-12
TCL1B	1.027362682	8.70E-14	1.02E-12
HSP90AA1	-1.062069964	8.77E-14	1.03E-12
KLRB1	1.008485876	8.85E-14	1.04E-12
DCAF4	1.006728607	1.06E-13	1.22E-12
IFIT1	-1.065116506	1.17E-13	1.33E-12
DGKE	1.309014003	1.20E-13	1.35E-12
ZNF91	1.100938798	1.40E-13	1.54E-12
ISG15	-1.383394406	1.40E-13	1.54E-12
PPT1	-1.244723046	1.43E-13	1.56E-12
BBS1	1.591094111	1.43E-13	1.56E-12
IPO9	1.12917642	1.52E-13	1.65E-12
CACNA1E	2.100794162	1.57E-13	1.69E-12
ZNF24	1.037999711	1.57E-13	1.69E-12
MOGS	-1.035880566	1.61E-13	1.73E-12
UTRN	1.007780687	1.64E-13	1.76E-12
SOX18	1.103763317	1.82E-13	1.93E-12
OVGP1	1.212920592	1.84E-13	1.95E-12
TREM1	-1.204285995	1.84E-13	1.95E-12

CD14	-1.215897328	1.86E-13	1.96E-12
S100A6	-1.613944441	1.92E-13	2.02E-12
TOE1	1.128435332	1.95E-13	2.04E-12
PUS7L	2.07818082	1.97E-13	2.06E-12
EIF1	-1.425766733	2.12E-13	2.18E-12
VCP	-1.184433098	2.12E-13	2.18E-12
FGR	-1.099912614	2.17E-13	2.22E-12
FAM111A	1.133000214	2.27E-13	2.32E-12
C1QB	-1.311167766	2.30E-13	2.34E-12
SFXN1	1.596447777	2.37E-13	2.41E-12
TUBB2A	-1.014121955	2.49E-13	2.52E-12
SLA	-1.144694729	2.51E-13	2.53E-12
BDH1	1.163132373	2.51E-13	2.53E-12
SCAI	1.265356467	2.53E-13	2.54E-12
ADRA1D	1.3398716	2.67E-13	2.66E-12
MRPS27	1.201680339	2.70E-13	2.69E-12
LY9	1.143303052	2.74E-13	2.71E-12
PIP5K1B	1.128956298	2.85E-13	2.78E-12
BAG1	-1.071775677	3.24E-13	3.10E-12
LMF1	1.440664037	3.38E-13	3.23E-12
CRIP1	-1.118693571	3.40E-13	3.24E-12
C1orf21	1.284167591	3.41E-13	3.24E-12
MYCN	1.168539927	3.46E-13	3.28E-12
FOXE1	1.486895162	3.50E-13	3.32E-12
ZNF358	1.255763649	3.75E-13	3.53E-12
CYB5R3	-1.23626288	3.82E-13	3.59E-12
OFD1	1.003150775	3.94E-13	3.69E-12
GAS2	1.080305426	3.98E-13	3.72E-12
ALDH2	-1.211641681	4.00E-13	3.73E-12
GAA	-1.158790467	4.12E-13	3.81E-12
ASF1A	1.570991991	4.27E-13	3.95E-12
GATAD1	1.060099147	4.30E-13	3.97E-12
SIDT2	-1.19525066	4.34E-13	3.99E-12
ATP1B3	-1.121609062	4.34E-13	4.00E-12
GPR12	1.31747408	4.38E-13	4.02E-12
TMEM59	-1.009168863	4.41E-13	4.04E-12
TMEM97	1.185459229	4.45E-13	4.07E-12
TAF1D	1.253557669	4.51E-13	4.11E-12
PSMB10	-1.075011232	4.78E-13	4.33E-12
TSPO	-1.308569466	4.93E-13	4.45E-12
KRTAP5-9	1.161521678	4.94E-13	4.45E-12
MMP15	1.21025419	5.18E-13	4.63E-12
SOCS7	1.483447422	5.35E-13	4.77E-12
IRX4	1.161445121	5.42E-13	4.81E-12

STAT3	1.043310857	5.56E-13	4.92E-12
B2M	-1.073511957	5.65E-13	4.97E-12
PAIP2B	1.018487586	6.38E-13	5.51E-12
GPR20	1.146229943	6.48E-13	5.56E-12
PDZD7	1.082905334	6.61E-13	5.62E-12
VPRBP	1.231514035	6.65E-13	5.65E-12
GPR37	1.493207579	6.69E-13	5.68E-12
WWOX	1.228758828	6.78E-13	5.75E-12
PALM	1.275023079	6.82E-13	5.75E-12
PECAM1	-1.02976642	7.09E-13	5.91E-12
KRIT1	1.633881587	7.40E-13	6.11E-12
GPX7	1.149617755	7.41E-13	6.11E-12
KIR2DL2	1.110972091	7.47E-13	6.15E-12
C21orf91	1.247546824	8.14E-13	6.62E-12
GOLGA8A	1.319470268	8.39E-13	6.80E-12
PYHIN1	1.326566424	8.62E-13	6.92E-12
DUSP2	1.222713101	8.70E-13	6.95E-12
GPX1	-1.208309284	8.80E-13	7.03E-12
FUT5	1.258531369	8.83E-13	7.04E-12
PASK	1.035591138	9.27E-13	7.34E-12
GPR126	1.388774575	9.83E-13	7.77E-12
CAMLG	1.7122011	9.98E-13	7.83E-12
ZNF506	1.525324655	1.01E-12	7.90E-12
VPS39	1.11362518	1.03E-12	8.05E-12
ZNF43	1.0060611	1.08E-12	8.34E-12
NELL2	1.023391813	1.08E-12	8.35E-12
CRTAP	1.395952274	1.08E-12	8.37E-12
RAP1B	-1.062182653	1.15E-12	8.77E-12
TMEM194A	1.010873567	1.17E-12	8.92E-12
GZMA	1.250968588	1.21E-12	9.16E-12
UBB	-1.494849146	1.23E-12	9.33E-12
ATP1B1	-1.07124619	1.26E-12	9.49E-12
OAZ1	-1.52085958	1.30E-12	9.75E-12
ARPC2	-1.309933611	1.31E-12	9.81E-12
TKT	-1.354749091	1.31E-12	9.83E-12
UBOX5	1.038999907	1.32E-12	9.89E-12
LAMP1	-1.207944545	1.34E-12	9.99E-12
DNPEP	1.052681345	1.35E-12	1.01E-11
ULK4	1.118203659	1.36E-12	1.01E-11
CNPY4	1.483890832	1.37E-12	1.02E-11
GNAI2	-1.325769876	1.39E-12	1.03E-11
FHIT	1.066254037	1.40E-12	1.04E-11
QRSL1	1.313009199	1.41E-12	1.04E-11
ATP5E	-1.457774717	1.42E-12	1.05E-11

MAPKAPK5	1.063995019	1.44E-12	1.06E-11
NPR2	1.16955401	1.45E-12	1.06E-11
POMT2	1.011787178	1.47E-12	1.08E-11
ATP8B2	1.19095219	1.47E-12	1.08E-11
N4BP3	1.999899862	1.49E-12	1.09E-11
ANXA1	-1.203821033	1.49E-12	1.09E-11
ZNF507	1.014814481	1.50E-12	1.09E-11
INTS6	1.11673532	1.53E-12	1.11E-11
EFNB3	1.332840222	1.60E-12	1.15E-11
ARG1	1.282341879	1.61E-12	1.16E-11
KRR1	1.672196535	1.68E-12	1.20E-11
TCF7L2	1.066610491	1.69E-12	1.21E-11
GNG5	-1.001269992	1.75E-12	1.24E-11
BCR	1.060148354	1.80E-12	1.27E-11
ANXA2	-1.21536265	1.81E-12	1.27E-11
TBC1D19	1.014830949	1.81E-12	1.27E-11
ACVR2B	1.331761077	1.86E-12	1.30E-11
GAMT	1.544761027	1.88E-12	1.31E-11
LGR4	1.622363547	2.02E-12	1.40E-11
FLJ11710	1.019278118	2.03E-12	1.40E-11
MAB21L2	1.263481317	2.13E-12	1.46E-11
ZNF510	1.307549136	2.17E-12	1.49E-11
RETN	-1.13155778	2.32E-12	1.58E-11
FEM1C	1.073150317	2.49E-12	1.69E-11
CA4	1.133660654	2.63E-12	1.76E-11
KCNK7	1.630949213	2.68E-12	1.80E-11
E2F5	1.09817324	2.78E-12	1.85E-11
OSGEPL1	1.170014517	2.81E-12	1.87E-11
ZNF609	1.025202616	3.02E-12	1.98E-11
COX6B1	-1.038994676	3.03E-12	1.98E-11
LOC202181	1.508011291	3.06E-12	2.00E-11
FAM168A	1.166972648	3.07E-12	2.01E-11
INPP4B	1.026996966	3.09E-12	2.02E-11
RAD9A	1.389293478	3.12E-12	2.04E-11
HBS1L	1.1264736	3.24E-12	2.10E-11
OGDH	1.525852682	3.25E-12	2.11E-11
ARHGAP24	1.426664556	3.26E-12	2.11E-11
TRIM66	1.129827282	3.29E-12	2.12E-11
NBPF1	1.459869104	3.31E-12	2.14E-11
FLJ10038	1.04591295	3.33E-12	2.15E-11
ZNF430	1.352110381	3.35E-12	2.16E-11
PLA2G15	-1.251476721	3.39E-12	2.18E-11
MBTPS2	1.07347919	3.40E-12	2.18E-11
INADL	1.027082622	3.43E-12	2.19E-11

CLIC1	-1.150388597	3.55E-12	2.26E-11
CTRL	1.768029171	3.64E-12	2.31E-11
TMEM176B	-1.02396212	3.80E-12	2.40E-11
TNIK	1.034445565	3.82E-12	2.40E-11
DTX3	1.52198758	3.86E-12	2.42E-11
SEPT9	-1.234095294	3.91E-12	2.45E-11
ZAP70	1.511476039	3.97E-12	2.48E-11
PNPLA4	1.216806351	4.05E-12	2.53E-11
JAM3	1.323285165	4.12E-12	2.57E-11
GEMIN8	1.285726321	4.21E-12	2.62E-11
TCEB3	1.053651193	4.24E-12	2.63E-11
TRIB2	1.12481275	4.32E-12	2.68E-11
MALT1	1.522290851	4.36E-12	2.69E-11
ZNF665	1.058970618	4.55E-12	2.79E-11
RNF41	1.042308291	4.60E-12	2.82E-11
SCN3B	1.197729323	4.66E-12	2.85E-11
TUBA1B	-1.294670221	4.80E-12	2.93E-11
RGS10	-1.075703276	4.82E-12	2.94E-11
PTMA	-1.191675039	4.95E-12	3.00E-11
ADAMTS5	1.693260349	5.15E-12	3.11E-11
PFKM	1.013172029	5.16E-12	3.12E-11
RUFY3	1.396683545	5.22E-12	3.14E-11
PPP2CA	1.177096198	5.30E-12	3.19E-11
CR2	1.113098122	5.54E-12	3.31E-11
DDX50	1.168825172	5.56E-12	3.31E-11
CCT5	1.280126881	5.71E-12	3.40E-11
SEMA3B	1.035753898	5.72E-12	3.40E-11
GH2	1.416245355	5.83E-12	3.45E-11
IFT52	1.090409103	5.90E-12	3.49E-11
ANGPT1	1.050721444	6.10E-12	3.59E-11
ADCY9	1.247724956	6.12E-12	3.59E-11
PPP1R2	1.004484699	6.13E-12	3.60E-11
DLD	1.36937459	6.83E-12	3.96E-11
ST8SIA2	1.452553805	6.89E-12	3.99E-11
SLC7A6	1.439234671	6.96E-12	4.03E-11
COX8A	-1.189634099	7.12E-12	4.11E-11
TTC9	1.37346421	7.20E-12	4.15E-11
METAP1	1.019814073	7.32E-12	4.21E-11
CCDC9	-1.274844936	7.34E-12	4.22E-11
OGFR	-1.206393362	7.42E-12	4.27E-11
KIR2DS4	1.386055785	7.52E-12	4.31E-11
ZNF132	1.366061076	7.70E-12	4.40E-11
CLDN11	1.342086364	7.94E-12	4.51E-11
NT5E	1.541944973	8.03E-12	4.55E-11

SAMD9	1.445110918	8.07E-12	4.57E-11
NDRG3	1.219612114	8.10E-12	4.58E-11
ERCC2	1.199085084	8.19E-12	4.63E-11
HLTF	1.069932705	8.71E-12	4.89E-11
AVIL	1.217218532	8.87E-12	4.97E-11
NFATC4	1.234138961	9.27E-12	5.17E-11
BCAS4	1.288363186	9.48E-12	5.28E-11
LEF1	1.42726147	9.55E-12	5.31E-11
EPM2A	1.105373224	9.56E-12	5.31E-11
DYNC1LI2	1.025722708	9.58E-12	5.31E-11
CASP8	1.14050648	9.62E-12	5.32E-11
MSH3	1.297073151	9.64E-12	5.33E-11
HLA-DPB1	-1.483929605	9.92E-12	5.47E-11
OTUD3	1.115870423	9.92E-12	5.47E-11
FOXRED2	1.41606751	1.07E-11	5.86E-11
SLC17A4	1.630386301	1.08E-11	5.88E-11
PLG	1.00288733	1.12E-11	6.07E-11
MRS2	1.322538025	1.15E-11	6.19E-11
ZNHIT6	1.20796139	1.16E-11	6.25E-11
IFT57	1.49046074	1.17E-11	6.32E-11
ZNF79	1.152071757	1.17E-11	6.32E-11
VAV2	1.143265678	1.19E-11	6.38E-11
HHLA3	1.419757618	1.19E-11	6.39E-11
IL18R1	1.438284565	1.19E-11	6.39E-11
ARHGEF12	1.733636548	1.21E-11	6.45E-11
RALGPS2	1.711672303	1.23E-11	6.55E-11
NR4A1	1.112284052	1.27E-11	6.73E-11
CPN2	1.877607994	1.27E-11	6.74E-11
ELF5	1.435660335	1.28E-11	6.76E-11
MLEC	1.060220223	1.29E-11	6.83E-11
HNF1B	1.237978911	1.29E-11	6.83E-11
ZNF140	1.181487208	1.31E-11	6.90E-11
SLC4A5	1.347039366	1.33E-11	6.98E-11
ABCC4	1.521094057	1.33E-11	6.99E-11
LIMD2	1.29331572	1.34E-11	7.03E-11
SUPT3H	1.019195171	1.34E-11	7.04E-11
PRKRIR	1.222938693	1.36E-11	7.10E-11
DHDDS	1.073323736	1.41E-11	7.37E-11
RASL12	1.474714343	1.41E-11	7.38E-11
TBC1D8	1.227209732	1.44E-11	7.49E-11
KIF1A	1.248468617	1.46E-11	7.59E-11
POLH	1.200105447	1.54E-11	7.94E-11
CYP27A1	-1.093790052	1.60E-11	8.18E-11
SULT1B1	1.402825164	1.62E-11	8.26E-11

ZNF473	1.255334192	1.63E-11	8.29E-11
FRS3	1.168385603	1.64E-11	8.34E-11
VPREB1	1.871728489	1.70E-11	8.62E-11
NPBWR2	1.156773653	1.71E-11	8.63E-11
ENDOD1	1.185303195	1.72E-11	8.67E-11
SYCE1L	1.432368307	1.73E-11	8.71E-11
WDR46	1.000391662	1.75E-11	8.82E-11
RPS27L	-1.047890108	1.78E-11	8.92E-11
MFGE8	1.256138236	1.80E-11	9.02E-11
TIGD6	1.389083315	1.84E-11	9.19E-11
SNIP1	1.160648683	1.85E-11	9.21E-11
CA5A	1.360281215	1.86E-11	9.25E-11
FGF9	1.035048467	1.87E-11	9.31E-11
EPM2AIP1	1.261879294	1.90E-11	9.44E-11
ENO2	1.486298034	1.94E-11	9.60E-11
SAMD14	1.652107222	1.96E-11	9.66E-11
ZNF706	1.002950595	2.07E-11	1.01E-10
USP7	1.101453077	2.16E-11	1.05E-10
EHBP1	1.032446915	2.17E-11	1.05E-10
C10orf2	1.23387997	2.17E-11	1.05E-10
PPP2R5A	1.199656145	2.18E-11	1.06E-10
GIN1	1.056447795	2.20E-11	1.07E-10
PPBP	-1.123233756	2.24E-11	1.08E-10
SMURF2	1.604525776	2.31E-11	1.11E-10
CHGA	1.352596063	2.48E-11	1.18E-10
FUT2	1.373378939	2.54E-11	1.20E-10
SHC3	1.281384945	2.55E-11	1.20E-10
RGS6	1.284592309	2.61E-11	1.23E-10
KIAA0485	1.252308514	2.62E-11	1.23E-10
HEXIM1	1.024745003	2.62E-11	1.23E-10
ERI3	1.135056117	2.63E-11	1.23E-10
ZNF468	1.164195648	2.71E-11	1.26E-10
DTNB	1.210346987	2.82E-11	1.31E-10
AXIN1	1.284963251	2.82E-11	1.31E-10
LOC100130331	1.376568727	2.85E-11	1.32E-10
EFHC1	1.000735951	2.87E-11	1.33E-10
GHRHR	1.55170407	2.88E-11	1.33E-10
ZC3H13	1.489570897	2.88E-11	1.33E-10
C16orf3	1.353568415	2.94E-11	1.35E-10
RPS24	-1.162938702	2.94E-11	1.35E-10
PLCL1	1.534442733	3.06E-11	1.40E-10
TOB2	1.174172906	3.09E-11	1.41E-10
PLCH2	1.17357369	3.17E-11	1.44E-10
HEMK1	1.37488988	3.26E-11	1.47E-10

TM2D3	1.007500698	3.29E-11	1.49E-10
CYP2E1	1.202611687	3.40E-11	1.53E-10
TMEM8B	1.079457859	3.44E-11	1.55E-10
KCNIP2	1.345622747	3.54E-11	1.58E-10
HSPB1	-1.06342593	3.56E-11	1.59E-10
RPLP2	-1.449368866	3.56E-11	1.59E-10
HLA-DMA	-1.114177208	3.58E-11	1.60E-10
SCNN1B	1.501996847	3.67E-11	1.64E-10
RPS6KB1	1.115521079	3.71E-11	1.65E-10
KIRREL	1.073071725	3.76E-11	1.67E-10
HTR3A	1.433260458	3.81E-11	1.68E-10
SEC61A2	1.40626658	3.82E-11	1.68E-10
TJP1	1.191418344	3.83E-11	1.69E-10
DLX2	1.23989381	3.87E-11	1.70E-10
CAPRIN1	1.074414014	3.87E-11	1.70E-10
AGBL5	1.381136019	3.98E-11	1.75E-10
GRAMD1B	1.241914387	3.99E-11	1.75E-10
MYCT1	1.376460571	4.02E-11	1.76E-10
COX15	1.472555941	4.04E-11	1.77E-10
PF4V1	1.481693244	4.05E-11	1.77E-10
BIRC2	1.085513386	4.08E-11	1.78E-10
MAP4	-1.243758318	4.08E-11	1.78E-10
TXNIP	-1.032876772	4.13E-11	1.80E-10
SOX21	1.498292093	4.15E-11	1.81E-10
HIST3H2A	1.466885318	4.17E-11	1.81E-10
UBASH3A	1.208101218	4.20E-11	1.82E-10
ARMCX3	1.13650306	4.34E-11	1.88E-10
ADAMTS7	-1.195959573	4.48E-11	1.92E-10
XPO4	1.132923467	4.57E-11	1.96E-10
ESPN	1.31383044	4.65E-11	1.99E-10
PRDM11	1.001437256	4.66E-11	1.99E-10
EML3	1.028757191	4.72E-11	2.01E-10
FAM69A	1.20620017	4.72E-11	2.01E-10
LY6G5C	1.040763041	4.83E-11	2.06E-10
CSNK1A1	1.119673744	4.86E-11	2.07E-10
EPPK1	1.352449992	5.02E-11	2.13E-10
C6orf120	1.072779205	5.11E-11	2.16E-10
UBE2O	1.141231734	5.15E-11	2.17E-10
CASKIN2	1.017643899	5.27E-11	2.22E-10
CAMTA1	1.334803918	5.34E-11	2.24E-10
AP4M1	1.269433053	5.41E-11	2.27E-10
FBXO17	1.047454819	5.43E-11	2.27E-10
TPR	1.029306902	5.87E-11	2.43E-10
KIR3DL1	1.372738028	5.90E-11	2.44E-10

STRADA	1.043162645	5.90E-11	2.44E-10
CYP3A5	1.045285677	6.19E-11	2.54E-10
MTHFSD	1.030479407	6.55E-11	2.68E-10
RBM15B	1.105689368	6.56E-11	2.68E-10
ZNF222	1.078577057	6.58E-11	2.69E-10
SMC5	1.222730633	6.64E-11	2.71E-10
XPNPEP2	1.136276975	7.00E-11	2.82E-10
GM2A	-1.186619557	7.26E-11	2.91E-10
STK17A	1.027028962	7.53E-11	3.01E-10
EID1	1.04716086	7.54E-11	3.01E-10
FAM172A	1.148440798	7.56E-11	3.02E-10
ANKFY1	1.426515409	7.66E-11	3.06E-10
INPP1	1.13071146	7.71E-11	3.07E-10
MICAL3	1.281125973	7.71E-11	3.07E-10
MASP2	1.211813259	7.90E-11	3.14E-10
DRD2	1.119830446	7.92E-11	3.14E-10
IL21R	1.163171752	7.95E-11	3.15E-10
USP25	1.033217176	8.04E-11	3.18E-10
SAP18	1.050514307	8.04E-11	3.18E-10
GPR17	1.107712032	8.24E-11	3.25E-10
FGF4	1.375749731	8.34E-11	3.28E-10
T	1.401100783	8.45E-11	3.32E-10
ZNF638	1.074833922	8.46E-11	3.32E-10
PCLO	1.77537288	8.64E-11	3.38E-10
GALNT3	1.653231687	8.65E-11	3.38E-10
SYT11	1.180397862	8.72E-11	3.40E-10
SNX4	1.217657439	8.85E-11	3.45E-10
IRAK4	1.263645668	8.87E-11	3.46E-10
MED17	1.091813415	9.34E-11	3.63E-10
PTGDR2	1.594948269	9.43E-11	3.65E-10
KLK15	1.438808956	9.69E-11	3.75E-10
CCDC134	1.183799968	9.76E-11	3.77E-10
CCNT2	1.322356081	9.79E-11	3.78E-10
RAB22A	1.191087344	1.00E-10	3.85E-10
SAMD4B	1.462965042	1.01E-10	3.88E-10
VGF	1.099194724	1.06E-10	4.04E-10
GRPR	1.428163733	1.06E-10	4.05E-10
IL1R2	1.314459739	1.07E-10	4.06E-10
PNN	1.25817252	1.09E-10	4.13E-10
CSDC2	1.319933249	1.09E-10	4.15E-10
MOBP	1.398000828	1.10E-10	4.17E-10
MED1	1.375524977	1.11E-10	4.21E-10
ASB7	1.272281304	1.14E-10	4.31E-10
SLC16A7	1.194381153	1.19E-10	4.46E-10

CRK	1.094552534	1.20E-10	4.50E-10
HUS1	1.044577667	1.23E-10	4.60E-10
ACBD3	1.179097152	1.26E-10	4.68E-10
ALDH5A1	1.273600781	1.29E-10	4.79E-10
BCL7A	1.218228848	1.29E-10	4.80E-10
ALOXE3	1.032245829	1.30E-10	4.85E-10
KLHL22	1.130722856	1.32E-10	4.90E-10
PYY	1.102945967	1.33E-10	4.95E-10
CD80	1.338035562	1.36E-10	5.02E-10
C2orf72	1.113248914	1.38E-10	5.10E-10
LMOD1	1.420595208	1.41E-10	5.18E-10
HLA-DQB1	-1.033165337	1.44E-10	5.30E-10
CHMP2B	1.196523182	1.46E-10	5.37E-10
AIDA	1.219218499	1.55E-10	5.64E-10
NR1D2	1.03761556	1.55E-10	5.66E-10
PRDX2	1.087632183	1.62E-10	5.90E-10
NDUFA3	-1.070854312	1.65E-10	6.00E-10
EHD2	1.139755817	1.66E-10	6.03E-10
AHSG	1.362963497	1.67E-10	6.04E-10
ARL4D	1.15375831	1.67E-10	6.05E-10
ANKRD53	1.3593523	1.70E-10	6.13E-10
SLC14A1	1.277387577	1.70E-10	6.14E-10
CYP11B1	1.440557871	1.70E-10	6.16E-10
PBLD	1.01613183	1.74E-10	6.28E-10
SPTBN4	1.160228624	1.78E-10	6.42E-10
SORBS1	1.195154826	1.82E-10	6.52E-10
RAB14	1.401471389	1.83E-10	6.55E-10
CTAGE1	1.223519857	1.84E-10	6.59E-10
NAP1L4	1.136075814	1.86E-10	6.64E-10
FOXJ2	1.299546686	1.86E-10	6.65E-10
LATS1	1.014919763	1.90E-10	6.76E-10
C11orf16	1.166343582	1.96E-10	6.97E-10
WNT4	1.126130918	2.06E-10	7.27E-10
SIPA1L3	1.254458508	2.14E-10	7.50E-10
EP400	1.094354463	2.26E-10	7.86E-10
TMOD2	1.125311711	2.29E-10	7.97E-10
ARHGEF9	1.279791503	2.34E-10	8.14E-10
METTL9	1.5446314	2.36E-10	8.22E-10
LMAN1	1.152301964	2.39E-10	8.29E-10
NKX2-5	-1.24162692	2.50E-10	8.64E-10
SLC22A17	1.063927277	2.60E-10	8.92E-10
PER3	1.335591905	2.62E-10	8.98E-10
PHF8	1.146091559	2.63E-10	9.01E-10
CACNA2D3	1.187677684	2.73E-10	9.31E-10

CADM3	1.015875936	2.79E-10	9.49E-10
VIL1	1.003853203	2.83E-10	9.61E-10
DUSP7	1.00766684	2.96E-10	9.98E-10
NEUROG1	1.130644844	3.07E-10	1.03E-09
EFEMP2	1.181401201	3.10E-10	1.04E-09
PGGT1B	1.169018731	3.15E-10	1.05E-09
GPR3	1.019754262	3.18E-10	1.06E-09
MYBPC2	1.576418522	3.24E-10	1.08E-09
CST2	1.40779584	3.25E-10	1.08E-09
ANKRD12	1.081025095	3.32E-10	1.10E-09
NEFM	1.035217081	3.37E-10	1.12E-09
FGD1	1.485587717	3.45E-10	1.14E-09
MYL4	1.283371129	3.48E-10	1.15E-09
BZRAP1	1.274526339	3.51E-10	1.16E-09
CRABP1	1.440754664	3.52E-10	1.16E-09
POLR3F	1.031166876	3.55E-10	1.17E-09
DDX52	1.266958579	3.56E-10	1.17E-09
ACPP	1.111607109	3.58E-10	1.18E-09
CPEB1	1.214863929	3.67E-10	1.20E-09
PDE4D	1.157709313	3.74E-10	1.22E-09
TNKS	1.011117975	3.75E-10	1.23E-09
FOLR3	1.052092528	3.80E-10	1.24E-09
MGST3	-1.239121321	3.81E-10	1.24E-09
NTRK2	1.206500453	3.90E-10	1.27E-09
CDCA4	1.192881374	3.95E-10	1.28E-09
PPAT	1.144220926	4.06E-10	1.32E-09
GLP2R	1.026962687	4.06E-10	1.32E-09
EIF5A2	1.003306462	4.08E-10	1.32E-09
PBX1	1.061803288	4.11E-10	1.33E-09
INPP5A	1.080105367	4.20E-10	1.36E-09
IFNA4	1.085793115	4.25E-10	1.37E-09
TMEM135	1.265776809	4.28E-10	1.38E-09
TMCC2	1.147863466	4.42E-10	1.41E-09
PIGA	1.061384737	4.88E-10	1.54E-09
KIAA0319	1.413878448	5.07E-10	1.60E-09
LZTFL1	1.0012141	5.12E-10	1.61E-09
GAST	1.152537374	5.12E-10	1.62E-09
HP	-1.343053809	5.20E-10	1.64E-09
RSAD2	1.128472012	5.29E-10	1.66E-09
USP34	1.056421351	5.47E-10	1.71E-09
GP1BA	1.389186342	5.73E-10	1.79E-09
MCTP2	1.28131513	5.79E-10	1.80E-09
HOXB13	1.266375189	5.90E-10	1.83E-09
SCAMP4	1.022451073	5.94E-10	1.84E-09

TPSG1	-1.200736778	6.06E-10	1.88E-09
RPL41	-1.257128861	6.09E-10	1.88E-09
RPL19	-1.244305627	6.39E-10	1.97E-09
ZNF292	1.109182758	6.51E-10	2.01E-09
COX7A2	-1.017398659	6.53E-10	2.01E-09
HLA-DQA1	-1.176128659	6.70E-10	2.06E-09
RPLP1	-1.032933153	6.78E-10	2.08E-09
KLHL7	1.137165132	6.86E-10	2.10E-09
PRICKLE3	1.071404551	6.99E-10	2.14E-09
GATA1	1.433009102	7.12E-10	2.17E-09
KIF3C	1.419959618	7.25E-10	2.21E-09
COL6A3	1.129852192	7.37E-10	2.24E-09
GNG4	1.19309948	7.63E-10	2.31E-09
SNX16	1.492641327	7.65E-10	2.31E-09
DLG3	1.077672222	8.11E-10	2.44E-09
TNFRSF4	1.033491208	8.29E-10	2.49E-09
VAMP8	-1.225582572	8.36E-10	2.51E-09
DHTKD1	1.036437489	8.64E-10	2.58E-09
TRAM2	1.222855657	8.87E-10	2.64E-09
MBOAT2	1.523916641	9.02E-10	2.69E-09
TTC22	1.349761658	9.29E-10	2.75E-09
SLC25A5	-1.009060343	9.43E-10	2.79E-09
PXDN	1.003069321	9.57E-10	2.83E-09
TRIM2	1.188652219	9.75E-10	2.88E-09
TRIM33	1.119042178	9.88E-10	2.91E-09
ARTN	1.03267489	9.97E-10	2.93E-09
C6orf25	1.134447083	1.05E-09	3.06E-09
TLK1	1.009488521	1.19E-09	3.46E-09
RNF24	1.262686609	1.42E-09	4.04E-09
RPL11	-1.219681161	1.46E-09	4.13E-09
ECE1	1.000191001	1.58E-09	4.47E-09
HSPB6	1.107435111	1.71E-09	4.82E-09
KDM5A	1.084840785	1.72E-09	4.84E-09
STMN2	1.140718157	1.73E-09	4.85E-09
MADCAM1	1.007141539	1.76E-09	4.94E-09
GJB3	1.166798168	1.77E-09	4.96E-09
SENP6	1.034499486	1.80E-09	5.03E-09
OLFM4	1.291290952	1.95E-09	5.40E-09
ACTC1	1.070483308	2.07E-09	5.72E-09
FRMD4B	1.063369179	2.84E-09	7.65E-09
MAP3K9	1.039260676	2.90E-09	7.80E-09
SPTLC2	1.298713657	3.27E-09	8.69E-09
IRF2	1.013613522	3.49E-09	9.22E-09
PHTF1	1.06348797	3.49E-09	9.23E-09

CRYBB3	1.080897479	3.56E-09	9.40E-09
C8orf4	1.016745034	3.77E-09	9.90E-09
BCL10	1.193699325	3.82E-09	1.00E-08
MPL	1.18858773	4.11E-09	1.07E-08
BCAM	1.038412347	4.13E-09	1.08E-08
PDE5A	1.00349972	4.20E-09	1.09E-08
HRH3	1.044003859	4.26E-09	1.11E-08
HLA-DQB2	-1.107048361	4.44E-09	1.15E-08
GRAMD1C	1.079680188	4.74E-09	1.22E-08
ZFYVE9	1.123939227	5.37E-09	1.36E-08
BAGE	1.231988445	6.45E-09	1.61E-08
SSTR2	1.28588127	6.89E-09	1.70E-08
NKX2-8	1.11561984	8.43E-09	2.06E-08
RPL32	-1.047559196	8.66E-09	2.11E-08
RPS4X	-1.009338509	9.02E-09	2.19E-08
SIX3	1.051091361	9.32E-09	2.26E-08
PROP1	1.120731382	9.98E-09	2.40E-08
RPL23A	-1.053972749	1.15E-08	2.74E-08
STK38L	1.015233923	1.23E-08	2.90E-08
PTGS2	1.152049872	1.27E-08	3.01E-08
UPK3B	1.139213643	1.47E-08	3.44E-08
MAK	1.024987947	1.67E-08	3.85E-08
RPL27	-1.192552165	1.74E-08	4.00E-08
CPLX2	1.062829348	1.91E-08	4.36E-08
RPS14	-1.034803462	1.91E-08	4.37E-08
CD52	-1.190696792	1.92E-08	4.39E-08
SIK3	1.039294621	2.40E-08	5.39E-08
SLC39A6	1.05512665	2.76E-08	6.15E-08
FAM198B	1.028289209	2.87E-08	6.37E-08
USP12	1.024898801	2.91E-08	6.44E-08
RRAD	1.0451362	3.18E-08	7.01E-08
ADAM28	1.023393705	3.26E-08	7.18E-08
BAALC	1.007132478	3.53E-08	7.73E-08
F5	1.093471993	3.73E-08	8.14E-08
ITGB3	1.240047215	3.77E-08	8.22E-08
RPS10	-1.232847393	3.96E-08	8.59E-08
PRSS23	1.019053198	4.45E-08	9.58E-08
ATP1B2	1.106283728	5.39E-08	1.14E-07
KRT14	1.292763576	5.62E-08	1.19E-07
KRT13	1.027637649	5.95E-08	1.25E-07
RPL35A	-1.062971801	6.29E-08	1.32E-07
NOVA2	1.076109888	1.00E-07	2.04E-07
YTHDC1	1.053827494	1.36E-07	2.71E-07
RPS21	-1.104134994	1.46E-07	2.91E-07

STEAP4	1.06561068	3.42E-07	6.48E-07
SERPINB2	1.027916658	6.10E-06	9.96E-06
