# Supplementary material for: Identification of the Molecular Subgroups in Idiopathic Pulmonary Fibrosis by Gene Expression Profiles
Source: Comput Math Methods Med. 2021 Oct 4;2021:7922594. doi: 10.1155/2021/7922594 (PMC8505108; doi:10.1155/2021/7922594)
Supplement: Supplementary Materials — There are three supplementary materials in this article. [file 7922594.f1.zip › supplementary table 2.pdf]

supplementary table 2

Upregulating and downregulating DEGs

Upregulating DEGs	Downregulating DEGs
LRRN3	PLA2G7
EMG1	VCAN
TMEM204	SORL1
RPL23AP32	GSN
FAM153A	LHFPL2
ANKRD2	SPP1
KRTAP5-8	CTSK
POU3F3	TGFBI
GFAP	AQP9
APOL3	LITAF
BDH2	EMP1
BAG3	LYN
BACH2	ICAM3
MSX1	S100P
MS4A1	ITGAX
ZNF331	CD97
GDNF	CXCR4
KLRF1	PSAP
PRX	VASP
CD79B	VIM
SOX17	LIPA
TCF7	ARPC5
CTSW	SPRR1A
C19orf53	TNFAIP2
MST1	GCA
GPT	SERPINA1
MRPL40	SRGN
CLDN5	LY96
HPGD	IL1RN
MTG1	GRN
KLHDC2	TCIRG1
CLIC3	PRCP
S1PR1	C1orf54
ZNF862	LGALS1
ATP5S	LGALS3
HPCA	ATP6AP2
CXCR5	PLBD1
ALG13	HEBP2
PTCD2	FBP1

RIC3	CTSA
ZNF492	CD163
USP53	RAC2
CHRNB2	S100A11
NDUFAF4	QSOX1
CNR1	MSN
PHACTR4	CAPG
CACNB1	ABR
NKX3-1	PGD
PCDH9	ACP5
IRX5	GLA
SMA4	CTSZ
ABHD10	EMILIN2
CNTNAP2	CTSD
TSPYL2	HIST1H3C
FRMD4A	ARHGDIB
RNF144A	H3F3A
SPTBN1	ZFP36
TCL1B	MS4A4A
KLRB1	DAZAP2
DCAF4	ALOX5
DGKE	ATP6V0C
ZNF91	LCP1
BBS1	STAT6
IPO9	COTL1
CACNA1E	KRT81
ZNF24	MARCO
UTRN	FCGR1B
SOX18	VASH1
OVGP1	NPC2
TOE1	TYMP
PUS7L	SNX2
FAM111A	APOE
SFXN1	SGK1
BDH1	ACTB
SCAI	HSP90B1
ADRA1D	CSF1R
MRPS27	S100A8
LY9	CEBPB
PIP5K1B	TUBB6
LMF1	FCER1G
C1orf21	SH3BGRL3
MYCN	LMNA
FOXE1	HLA-B

ZNF358	S100A10
OFD1	CEBPD
GAS2	S100A4
ASF1A	CYP2W1
GATAD1	AIF1
GPR12	CDC42
TMEM97	SNX3
TAF1D	ATP6AP1
KRTAP5-9	GMFG
MMP15	HCLS1
SOCS7	ACADVL
IRX4	CHMP2A
STAT3	HMOX1
PAIP2B	HSP90AA1
GPR20	IFIT1
PDZD7	ISG15
VPRBP	PPT1
GPR37	MOGS
WWOX	TREM1
PALM	CD14
KRIT1	S100A6
GPX7	EIF1
KIR2DL2	VCP
C21orf91	FGR
GOLGA8A	C1QB
PYHIN1	TUBB2A
DUSP2	SLA
FUT5	BAG1
PASK	CRIP1
GPR126	CYB5R3
CAMLG	ALDH2
ZNF506	GAA
VPS39	SIDT2
ZNF43	ATP1B3
NELL2	TMEM59
CRTAP	PSMB10
TMEM194A	TSPO
GZMA	B2M
UBOX5	PECAM1
DNPEP	GPX1
ULK4	RAP1B
CNPY4	UBB
FHIT	ATP1B1
QRSL1	OAZ1

MAPKAPK5	ARPC2
NPR2	TKT
POMT2	LAMP1
ATP8B2	GNAI2
N4BP3	ATP5E
ZNF507	ANXA1
INTS6	GNG5
EFNB3	ANXA2
ARG1	RETN
KRR1	COX6B1
TCF7L2	PLA2G15
BCR	CLIC1
TBC1D19	TMEM176B
ACVR2B	SEPT9
GAMT	TUBA1B
LGR4	RGS10
FLJ11710	PTMA
MAB21L2	COX8A
ZNF510	CCDC9
FEM1C	OGFR
CA4	HLA-DPB1
KCNK7	CYP27A1
E2F5	RPS27L
OSGEPL1	PPBP
ZNF609	RPS24
LOC202181	HSPB1
FAM168A	RPLP2
INPP4B	HLA-DMA
RAD9A	MAP4
HBS1L	TXNIP
OGDH	ADAMTS7
ARHGAP24	GM2A
TRIM66	HLA-DQB1
NBPF1	NDUFA3
FLJ10038	NKX2-5
ZNF430	MGST3
MBTPS2	HP
INADL	TPSG1
CTRL	RPL41
TNIK	RPL19
DTX3	COX7A2
ZAP70	HLA-DQA1
PNPLA4	RPLP1
JAM3	VAMP8

GEMIN8
TCEB3
TRIB2
MALT1
ZNF665
RNF41
SCN3B
ADAMTS5
PFKM
RUFY3
PPP2CA
CR2
DDX50
CCT5
SEMA3B
GH2
IFT52
ANGPT1
ADCY9
PPP1R2
DLD
ST8SIA2
SLC7A6
TTC9
METAP1
KIR2DS4
ZNF132
CLDN11
NT5E
SAMD9
NDRG3
ERCC2
HLTF
AVIL
NFATC4
BCAS4
LEF1
EPM2A
DYNC1LI2
CASP8
MSH3
OTUD3
FOXRED2
SLC17A4
SLC25A5
RPL11
HLA-DQB2
RPL32
RPS4X
RPL23A
RPL27
RPS14
CD52
RPS10
RPL35A
RPS21

PLG
MRS2
ZNHIT6
IFT57
ZNF79
VAV2
HHLA3
IL18R1
ARHGEF12
RALGPS2
NR4A1
CPN2
ELF5
MLEC
HNF1B
ZNF140
SLC4A5
ABCC4
LIMD2
SUPT3H
PRKRIR
DHDDS
RASL12
TBC1D8
KIF1A
POLH
SULT1B1
ZNF473
FRS3
VPREB1
NPBWR2
ENDOD1
SYCE1L
WDR46
MFGE8
TIGD6
SNIP1
CA5A
FGF9
EPM2AIP1
ENO2
SAMD14
ZNF706
USP7

EHBP1
C10orf2
PPP2R5A
GIN1
SMURF2
CHGA
FUT2
SHC3
RGS6
KIAA0485
HEXIM1
ERI3
ZNF468
DTNB
AXIN1
LOC100130331
EFHC1
GHRHR
ZC3H13
C16orf3
PLCL1
TOB2
PLCH2
HEMK1
TM2D3
CYP2E1
TMEM8B
KCNIP2
SCNN1B
RPS6KB1
KIRREL
HTR3A
SEC61A2
TJP1
DLX2
CAPRIN1
AGBL5
GRAMD1B
MYCT1
COX15
PF4V1
BIRC2
SOX21
HIST3H2A

UBASH3A
ARMCX3
XPO4
ESPN
PRDM11
EML3
FAM69A
LY6G5C
CSNK1A1
EPPK1
C6orf120
UBE2O
CASKIN2
CAMTA1
AP4M1
FBXO17
TPR
KIR3DL1
STRADA
CYP3A5
MTHFSD
RBM15B
ZNF222
SMC5
XPNPEP2
STK17A
EID1
FAM172A
ANKFY1
INPP1
MICAL3
MASP2
DRD2
IL21R
USP25
SAP18
GPR17
FGF4
T
ZNF638
PCLO
GALNT3
SYT11
SNX4

IRAK4
MED17
PTGDR2
KLK15
CCDC134
CCNT2
RAB22A
SAMD4B
VGF
GRPR
IL1R2
PNN
CSDC2
MOBP
MED1
ASB7
SLC16A7
CRK
HUS1
ACBD3
ALDH5A1
BCL7A
ALOXE3
KLHL22
PYY
CD80
C2orf72
LMOD1
CHMP2B
AIDA
NR1D2
PRDX2
EHD2
AHSG
ARL4D
ANKRD53
SLC14A1
CYP11B1
PBLD
SPTBN4
SORBS1
RAB14
CTAGE1
NAP1L4

FOXJ2
LATS1
C11orf16
WNT4
SIPA1L3
EP400
TMOD2
ARHGEF9
METTL9
LMAN1
SLC22A17
PER3
PHF8
CACNA2D3
CADM3
VIL1
DUSP7
NEUROG1
EFEMP2
PGGT1B
GPR3
MYBPC2
CST2
ANKRD12
NEFM
FGD1
MYL4
BZRAP1
CRABP1
POLR3F
DDX52
ACPP
CPEB1
PDE4D
TNKS
FOLR3
NTRK2
CDCA4
PPAT
GLP2R
EIF5A2
PBX1
INPP5A
IFNA4

TMEM135
TMCC2
PIGA
KIAA0319
LZTFL1
GAST
RSAD2
USP34
GP1BA
MCTP2
HOXB13
SCAMP4
ZNF292
KLHL7
PRICKLE3
GATA1
KIF3C
COL6A3
GNG4
SNX16
DLG3
TNFRSF4
DHTKD1
TRAM2
MBOAT2
TTC22
PXDN
TRIM2
TRIM33
ARTN
C6orf25
TLK1
RNF24
ECE1
HSPB6
KDM5A
STMN2
MADCAM1
GJB3
SENP6
OLFM4
ACTC1
FRMD4B
MAP3K9

SPTLC2
IRF2
PHTF1
CRYBB3
C8orf4
BCL10
MPL
BCAM
PDE5A
HRH3
GRAMD1C
ZFYVE9
BAGE
SSTR2
NKX2-8
SIX3
PROP1
STK38L
PTGS2
UPK3B
MAK
CPLX2
SIK3
SLC39A6
FAM198B
USP12
RRAD
ADAM28
BAALC
F5
ITGB3
PRSS23
ATP1B2
KRT14
KRT13
NOVA2
YTHDC1
STEAP4
SERPINB2
