# Supplementary material for: Identification of the Molecular Subgroups in Idiopathic Pulmonary Fibrosis by Gene Expression Profiles
Source: Comput Math Methods Med. 2021 Oct 4;2021:7922594. doi: 10.1155/2021/7922594 (PMC8505108; doi:10.1155/2021/7922594)
Supplement: Supplementary Materials — There are three supplementary materials in this article. [file 7922594.f1.zip › supplementary table 3.pdf]

supplementary table 3

Enrichment analysis of KEGG pathway (part)

Cluster	Description	pvalue	geneID
blue	Autophagy-animal	1.2183013164782E-07	AKT3/ATG12/ATG2B/ATG3/ATG5/ATG9A/BCL2L1/BECN1/BNIP3/CAMKK2/CTSD/EIF2S1/HIF1A/ITPR1/KRAS/LAMP2/MAP2K2/MAP3K7/MAPK3/NRAS/PIK3C3/PIK3CA/PIK3R1/PPP2CA/PPP2CB/PRKAA1/PRKACB/PTEN/RAB1A/RB1CC1/RHEB/RPS6KB1/RRAGD/RRAS/SH3GLB1/TANK/TBK1/TSC2/ULK2
greenyellow	Oxidative phosphorylation	1.20575136303187E-11	COX7A2/NDUFA4/NDUFA8/NDUFAB1/NDUFB1/NDUFB2/NDUFB4/NDUFB8/NDUFC1/NDUFS3/NDUFS6/UQCRFS1/UQCRQ
grey	ECM-receptor interaction	0.000286418592072073	CD44/COL4A1/COL6A1/COL6A3/GP1BA/ITGA2B/ITGB3/LAMA1/LAMA3/LAMA4/LAMA5/SDC4/THBS1
magenta	Ribosome	1.97412696775556E-77	FAU/RPL10A/RPL10L/RPL11/RPL12/RPL14/RPL15/RPL19/RPL22/RPL23A/RPL24/RPL27/RPL27A/RPL29/RPL3/RPL30/RPL31/RPL32/RPL34/RPL35/RPL35A/RPL36AL/RPL37/RPL37A/RPL38/RPL39/RPL41/RPL6/RPL7/RPL8/RPL9/RPLP0/RPS10/RPS11/RPS12/RPS14/RPS15A/RPS16/RPS17/RPS18/RPS19/RPS21/RPS23/RPS24/RPS25/RPS27/RPS27A/RPS27L/RPS28/RPS29/RPS3/RPS4X/RPS6/RPS7/RPS8
pink	Neuroactive ligand-receptor interaction	1.78903809480975E-14	ADRA1D/ADRB3/CHRNA4/CNR1/CORT/CRHR1/CRHR2/CSH1/DRD2/DRD4/GABRB2/GABRG2/GALR3/GH2/GHRHR/GHSR/GIPR/GLP2R/GNRH2/GRID2/GRIK5/GRIN1/GRIN2D/GRPR/HCRT/HCRTR1/HRH3/HTR1B/KISS1/LTB4R2/NPBWR2/OPRD1/OPRL1/OXT/OXTR/P2RX2/P2RX3/PPY/PRLH/PRSS2/PRSS3/PYY/SSTR2/SSTR3/TAAR2/TAAR5/TACR2/TBXA2R/TSHB
pink	Calcium signaling pathway	0.0012357790149814	ADRA1D/ADRB3/CACNA1B/CACNA1C/CACNA1E/CACNA1H/FGF3/GRIN1/GRIN2D/GRPR/HRC/LTB4R2/MST1/NOS

			1/NOS3/NTRK2/OXTR/P2RX2/P2RX3/TACR2/TBXA2R
tan	Th1 and Th2 cell differentiation	2.17064437565144E-07	CD247/CD3D/HLA-DMB/IL2RB/JAK3/RUNX3/STAT4/ZAP70
